# Ferroptosis: the potential value target in atherosclerosis

**DOI:** 10.1038/s41419-021-04054-3

**Published:** 2021-08-10

**Authors:** Siyu Ouyang, Jia You, Chenxi Zhi, Pin Li, Xiaoyan Lin, Xiaoqian Tan, Wentao Ma, Liang Li, Wei Xie

**Affiliations:** 1 Clinical Anatomy & Reproductive Medicine Application Institute, University of South China, Hengyang, 421001 Hunan China; 2 Institute of Cardiovascular Research, Key Laboratory for Atherosclerology of Hunan Province, Medical Research Center, Hunan Province Cooperative Innovation Center for Molecular Target New Drug Study, University of South China, Hengyang, 421001 Hunan China; 3 School of Public Health, University of South China, Hengyang, 421001 Hunan China

**Keywords:** Cell death, Cardiovascular diseases

## Abstract

In advanced atherosclerosis (AS), defective function-induced cell death leads to the formation of the characteristic necrotic core and vulnerable plaque. The forms and mechanisms of cell death in AS have recently been elucidated. Among them, ferroptosis, an iron-dependent form of necrosis that is characterized by oxidative damage to phospholipids, promotes AS by accelerating endothelial dysfunction in lipid peroxidation. Moreover, disordered intracellular iron causes damage to macrophages, vascular smooth muscle cells (VSMCs), vascular endothelial cells (VECs), and affects many risk factors or pathologic processes of AS such as disturbances in lipid peroxidation, oxidative stress, inflammation, and dyslipidemia. However, the mechanisms through which ferroptosis initiates the development and progression of AS have not been established. This review explains the possible correlations between AS and ferroptosis, and provides a reliable theoretical basis for future studies on its mechanism.

## FACTS

1. Endothelial cell ferroptosis is an initiating factor in atherosclerosis.
2. Gpx4 is a key player in the regulation of ferroptosis and atherosclerosis.
3. Increased lipid peroxides caused by ox-LDL accumulation within atherosclerotic plaques is a dominant condition for the onset of ferroptosis.

## OPEN QUESTIONS

1. Where are the sources of iron that influence atherosclerosis and how is the excess iron metabolized when ferroptosis occurs in atherosclerosis-related cells?
2. What is the relationship between lipid peroxidation that causes ferroptosis and those formed by ox LDL in atherosclerotic plaques?
3. Does the interplay between autophagy and ferroptosis have any effect on atherosclerosis?
4. Does ferroptosis-induced cell death affect the whole process of atherosclerosis?
5. Is there a relationship between iron metabolism and lipid metabolism in atherosclerosis?

## Introduction

Ferroptosis is a regulated form of cell death attributed to abundant cellular iron levels that lead to an imbalance in the production and clearance of lipid peroxides [1]. It is induced by erastin and ras-selective lethal small molecule 3 (RSL3) while its prevention is by iron chelators [2]. Ferroptosis is enhanced by the accumulation of lipid peroxides due to Fe^2+^-mediated Fenton reactions and reactive oxygen species (ROS) [3]. Glutathione peroxidase 4 (GPX4), a major lipid repair enzyme, scavenges for toxic lipids to block ferroptosis [1, 4]. AS is a type of peripheral vascular disease associated with toxic lipid accumulation in the walls of medium and large-sized arteries [5]. The pathogenesis of AS also involves dysregulated iron metabolism [6], decreased glutathione peroxidase (GPX) levels [7], and increased ROS [8] in AS-associated cell macrophages, VSMCs, and endothelial cells [9–11]. Notably, it has been preliminarily proved that ferroptosis mediates angiogenesis in AS [12]. Thus, this review further elucidates on the relationship between ferroptosis and AS-related risk factors and regulators, which may be harnessed using therapeutics to improve lesion regression.

## Iron-dependent ferroptosis and AS

### Effects of iron transport and storage on AS

Ferritin is the main iron storage protein in the cell. It is composed of a spherical shell cavity structure formed by two subunits, heavy chain and light chain [13]. The heavy chain exhibits ferrous oxidase activities that oxidize Fe^2+^ to Fe^3+^ which is then stored in ferritin to avoid the oxidative stress attributed to Fe^2+^-mediated Fenton reactions, and maintain intracellular iron homeostasis to prevent cellular damage and death. Upon excessive degradation of ferritin, elevated intracellular labile iron levels enhance sensitivity to ferroptosis [14], implying that ferritin regulates ferroptosis by binding excess iron to avoid oxidative damage [15].

Ferritin-mediated iron homeostasis plays a beneficial role in the cardiovascular system [16, 17]. Ferritin is a biomarker for non-fatal cardiovascular disease (CVD), especially among individuals with hyperlipidemia [18]. Elevated plasma ferritin concentration is a biomarker for early coronary heart disease (CHD), while abnormally elevated ferritin levels promote early atherogenesis and its associated complications [19–21]. These effects are attributed to dysregulated iron homeostasis [15, 21]. Iron metabolism can also be used to monitor the risk of AS in offspring. In a survey of carotid intima-media thickness (cIMT) among children, circulating ferritin levels were independently correlated with the changes in carotid intima-media thickness, especially among children whose father exhibited higher ferritin levels [22]. Surprisingly, Mendel’s random analysis showed that an increase in ferritin saturation following an increase in serum iron levels was correlated with a decrease in CHD risk [23], which may be attributed to the possibility of reverse causality bias. Ferritin and LDL cholesterol exert a synergistic effect on CVD [24].

Transferrin receptor 1 protein (TFR1), a crucial ferroptotic protein that accelerates iron uptake and ferritin synthesis, is significantly accumulated in the nuclear regions of many foamy cells. In atherosclerotic lesions, it contributes to the development and rupture of human carotid atheroma [25, 26]. Therefore, ferritin and TFR1 are important ferroptotic targets for the prevention of AS.

### Hepcidin regulated ferroportin affects AS

Hepcidin, a hormonal regulator of iron homeostasis, accelerates erastin-induced ferroptosis by increasing intracellular iron levels. This is attributed to the conformational change and degradation of ferroportin (FPN) [27, 28]. In the human aortic wall, hepcidin activates the TLR4/NF-κB pathway to initiate hepcidin autocrine-induced iron retention in murine macrophages [9], and enhances inflammation-mediated AS [29]. In a murine model, overexpressed *Hamp*, the gene encoding hepcidin, was shown to enhance the development of AS [30, 31]. Hamp^−/−^Ldlr^−/−^ mice exhibited low iron levels in aortic macrophages and decreased aortic macrophage activities [30]. In addition, interleukin-6 (IL-6)-induced overexpression of hepcidin is involved in immune responses and promotes AS [32–34]. However, it has not been established whether IL-6 affects iron metabolism through hepcidin to enhance AS progression.

## Effects of ferroptosis-associated ROS on AS

Elevated ROS levels enhance cell sensitivity to ferroptosis. This is attributed to elevated intracellular iron concentration, depletion of the antioxidant glutathione (GSH), or excessive AS inducer ox-LDL, which result in the accumulation of intracellular lipid peroxides [1, 35]. In the cardio-cerebral vascular system, ROS-induced phospholipid oxidation is mainly upregulated during ischemia/reperfusion (I/R) injury, which is an acute vascular ischemic event that is caused by atherosclerotic plaques, such as coronary artery stenosis and ischemic attack [36–39]. Therefore, the accumulation of ROS in AS pathologies can affect phospholipid metabolism [40, 41]. To a certain extent, elevated lipid-based ROS-induced ferroptosis has been implicated in pathophysiological processes of AS [1, 12].

### Ferroptosis-associated lipoxygenases (LOXs) mediate AS

Lipoxygenases (LOXs), including 15-lipoxygenase (15-LOX) and 12-lipoxygenase (12-LOX) are the key enzymes for ROS production. They are mainly expressed in macrophages after induction by IL-4, LPS, and hypoxia [42]. The overexpression of LOXs enhances ferroptosis and AS pathogenesis [43, 44]. Inhibition of 15-LOX significantly suppressed ox-LDL deposition in the subendothelial space and attenuated AS development. This is because it was mainly engaged in the synthesis of bioactive lipid mediators [44]. Moreover, 15-LOX inhibitors prevent erastin, RSL3, and arachidonate-15-lipoxygenase (ALOX15) induced ferroptosis, which is correlated with increased formation of lipid peroxides [2, 45]. *ALOX15B* was found to encode for an enzyme associated with the development of atherosclerotic plaques in humans and in a mouse model of hypercholesterolemia. It is involved in intracellular cholesterol metabolism, such as that of cholesterol intermediates desmosterol, lanosterol, 24, 25-dihydrolanosterol, and oxysterols in interleukin-4(IL-4)-stimulated macrophages [46]. Erastin-induced ferroptosis can be inhibited by *ALOX15B* silencing [45]. Similar to ALOX15, ALOX12 exhibits higher methylation levels in AS plaques, especially in endothelial cells [47], which is essential for p53-mediated ferroptosis after stress [48]. The implicated LOXs are closely correlated with ferroptosis-mediated AS. More studies are needed to determine whether LOXs directly lead to cell ferroptosis in atherosclerotic plaques and whether they affect lesion formation.

### ROS-induced oxidized phospholipids in AS

Oxidized phospholipid (OxPL), an ROS-induced product, enhances the risk factors for AS, including endothelial dysfunction [49], foam cell formation [50], abnormal proliferation, and ectopic migration of VSMCs [53]. It also acts as a proinflammatory mediator in atherosclerotic lesions [51, 52]. Studies have shown that OxPL combinate with immunoglobulin M (IgM) natural antibody E06 significantly inhibited AS in Ldlr^−/−^ mice, which appeared in the ameliorated aortic valve gradients and the decreased aortic valve calcification, by blocking ox-LDL uptake and suppressing the pro-inflammatory property of OxPL in macrophages [51]. These findings confirm the major proatherogenic role of OxPL. The accumulation of phospholipid hydroperoxides caused by GPx4 catalytic barrier in the endoplasmic reticulum (ER) is involved in ferroptosis [53, 54]. The phospholipid metabolism disorder may be a bridge between ferroptosis and AS. Elucidation of the roles of phospholipids in the pathogenesis of ferroptosis-induced AS will establish the relationship between AS and ferroptosis, and they could be potential disease biomarkers and novel therapeutic targets in AS-CVD.

### Mono-unsaturated fatty acids regulate lipid ROS-induced ferroptosis and AS

At the plasma membrane, mono-unsaturated fatty acids (MUFAs) suppress lipid ROS accumulation and inhibit ferroptosis in an acyl-CoA synthetase long-chain 3 (ACSL3) dependent manner [55]. In addition, supplementation with dietary MUFA was shown to ameliorate glycolipid metabolism as well as inflammation and inhibited AS development in Ldlr^−/−^ mice [56]. However, MUFA did not prevent AS in apolipoprotein E knockout (apoE^−/−^) mice [57]. The reason for this phenomenon may be that the LDL receptor-related proteins mainly mediate lipoprotein clearance by the liver when LDL receptors are absent, while the lack of apoE prevents the normal diet-induced increase of very-low-density lipoprotein (VLDL) production [58, 59]. Even though both AS and ferroptosis are impacted by MUFA, it has not been established whether MUFA mediated AS inhibition is associated with ferroptosis.

## The novel anti-ferroptosis role of GPx4 in eliminating lipid peroxidation in AS

Once selenoproteins are overexpressed, vascular endothelium damage can be prevented in AS-associated cardiovascular diseases [60]. Therefore, selenoproteins are involved in AS formation and development. Mutations in selenoprotein genes constitute a risk factor for peripheral AS [61]. Glutathione peroxidases (GPx), including GPx1 and GPx4, are mammal selenoproteins that protect cells from oxidative reactions, and prohibit inflammatory responses as well as oxidant-induced cell death [62]. Among them, a *GPx4* variant in the rs713041T allele enhances the risks for aortoiliac occlusive disease (AOID) and peripheral arterial disease (PAD) that result in atherosclerotic occlusions [61]. Age-associated decrease in *GPx4* expression has shown that it is an important predictor of AS [63]. Intuitively, GPx4, a hydroperoxide scavenger responsible for converting lipid hydroperoxides into non-toxic lipid alcohols [64, 65], effectively suppresses lipid hydroperoxides, including phospholipids, fatty acids as well as cholesterols, decreases vascular endothelial cell damage to oxidized lipids, and inhibits AS development. However, GPx1 does not have the same effects as GPx4 [66, 67]. Therefore, GPx4 plays a more important atheroprotective role than GPx1 in AS.

In addition to being a unique intracellular antioxidant enzyme [64, 68], GPx4 exerts resistance to ferroptosis by directly clearing the peroxidized phospholipids located in the membrane [64], diminishing hydroperoxy groups of complex lipids and extinguishing lipoxygenases. Experimentally, *GPx4* knockdown in a mesenchymal cell-line was highly correlated with sensitivity to ferroptosis [69], while *GPx4*-deficient T cells rapidly accumulated lipid peroxides, leading to ferroptosis [70]. These findings imply that GPx4 plays an important ferroptotic role in the pathophysiologic process of AS [7].

Selenium (Se), an essential component of selenoproteins, plays a crucial role in AS by mediating GPx4 expression. Deficiency in Se was shown to inhibit enzyme activity and mRNA expression levels of cytosolic GPx, thereby increasing lipid peroxidation in bovine arterial endothelial cells (BAEC) [71]. However, Se supplementation enhanced GPx4 expression as well as activity and inhibited oxidative stress in vascular endothelial cells or VSMCs [72, 73]. As a component of GPx4, Se is required to prevent hydroperoxide-induced ferroptosis [4]. In the presence of sufficient Se, antioxidants that upregulate GPx4 can potentially reverse lipid peroxidase-mediated atherogenic processes [74]. Statins such as fluvastatin are widely used as lipid regulating drugs in AS. They inhibit selenoprotein biosynthesis by preventing the production of isopentenyl pyrophosphate through the mevalonate pathway [69]. Fluvastatin was shown to suppress GPx4 expression in a time- and concentration-dependent manner, and exerted synergistic effects with the direct GPx4 inhibitor, RSL3 [75]. In conclusion, Se-mediated GPx4 affects the development of AS, and this process is closely associated with ferroptosis.

## Ferroptotic cells are involved in AS

### Ferroptosis-induced endothelial dysfunction aggravates AS

Endothelial dysfunction is an initial event in AS. Chronic iron overload leads to endothelial dysfunction through ROS and cyclooxygenase pathways that enhance the progression of AS in apoE^−^^/−^ mice, which may be attributed to an imbalance in diastolic and contractile factors synthesized by damaged VECs [6, 10]. Human umbilical vein endothelial cells (HUVEC) treated with erastin exhibited a rapid generation of ROS and a reduced viability. This process could be reversed by ferrostatin-1 (fer-1) [76]. Furthermore, there is direct evidence that VEC ferroptosis promotes AS by accelerating endothelial dysfunction during ROS-mediated lipid peroxidation [12]. This implies that erastin-induced VEC ferroptosis leads to endothelial dysfunction which is a risk factor for AS.

ox-LDL induced VEC damage, which promotes AS, is associated with ferroptosis. Mouse aortic endothelial cells (MAECs) treated with ox-LDL or erastin had elevated ROS, lipid peroxidation, and malondialdehyde (MDA) levels within the damaged mitochondria. However, fer-1 could suppress the generation of these peroxidation products, implying that ox-LDL can induce ferroptosis in MAECs. Inhibition of ferroptosis ameliorated ox-LDL-induced endothelial cell injury and lipid peroxidation [12]. Fer-1 and iron chelator deferoxamine mesylate, which plays the anti-ferroptotic role, rescued ferroptotic damage in endothelial cells and restored antioxidant activity as well as iron metabolism [77].

Activating transcription factor 3 (ATF3) promotes lipid peroxidation induced ferroptosis by suppressing system Xc^−^ to depleting intracellular GSH [78]. Surprisingly, ATF3 was found to be significantly overexpressed in macrophages and endothelial cells of human vascular walls with atherosclerotic plaques, but was barely detectable in the non-atherosclerotic artery. It was explicated in TUNEL-positive dead cells [79]. These findings imply that macrophagic and endothelial cell death in atherosclerotic plaques caused by ATF3 overexpression may be correlated with ferroptosis. However, these results contrast with those found in atherosclerotic plaques of apoE^−/−^ mice. The overexpression of ATF3, which is mainly expressed in macrophages and less in endothelial cells, was shown to improve atherosclerotic plaques by inhibiting phosphatidylinositol 3-kinase (PI3K)-matrix metalloproteinase 3 (MMP3) signal pathway, reducing elastic lamina damage, increasing plaque stability [80], and protecting cells from Toll-like receptor (TLR)-induced inflammation in vitro and in vivo [81]. Excluding species differences, this implies that elevated expression of ATF3 in human AS may be due to the increase in the body’s own protective responses rather than ferroptosis. Based on the current studies, we cannot be certain as to whether ATF3-regulated endothelial cell death at the transcriptional level promotes ferroptosis mediated AS in response to atherogenic agents.

### Associations between macrophage ferroptosis and AS

#### Ferroptosis influences the inflammatory phenotype in macrophages

AS refers to a chronic inflammatory pathogenesis in the intima of the large and medium-sized arteries, where the infiltrated inflammatory cells and macrophages are activated by cytokines and oxidative stress [82]. It has been documented that Fe promotes lipid peroxidation and GSH disulfide/total GSH ratio in THP-1 macrophages [83]. Macrophages derived from granulocyte-macrophage hematopoietic progenitor cells (GM-HPCs), may be damaged by ferroptosis through the NADPH oxidase 4 (NOX4)-ROS-P38-MAPK signaling pathway [84, 85]. Once there is an iron overload and exposure to ox-LDL and lipopolysaccharide (LPS)/interferon-γ (IFN-γ) [86], the number of M1 proinflammatory macrophages phenotype as well as inflammatory responses increase [87]. However, compared to M2 macrophages, M1 macrophages exert higher resistance to pharmacologically induced ferroptosis in vivo. This is because M1 macrophages maintain a higher level of intracellular iron, while M2 macrophages metabolize heme iron through the action of heme oxygenase 1 (HMOX1). The resistance of M1 and the sensitivity of M2 macrophages to RSL3 induced ferroptosis are associated with inducible NO synthase/NO•, which substitutes GPX4 as an anti-ferroptotic material and inhibits pro-ferroptotic lipid peroxidation [88]. Ferroptosis of macrophages can occur in advanced plaques [89], suggesting that ferroptosis of the polarized macrophages accelerates AS progress.

#### Macrophage associated iron metabolism affects AS

Macrophage is a key cell type in atherosclerotic plaques, where macrophage accumulation and iron deposition are displayed [90, 91]. However, the precise mechanisms by which iron levels in macrophages contribute to the pathogenesis of AS have not been established. Excess iron activates the generation of ROS to induce lipid peroxidation in foam cell-derived macrophages, leading to the instability of atherosclerotic plaques [92]. Moreover, iron metabolism disorders in macrophages are involved in inflammatory responses that aggravate the severity of AS [14], which may be closely associated with iron overload to induce macrophage polarization towards the M1 proinflammatory phenotype through the ROS/acetyl-p53 pathway [93, 94]. Furthermore, through the nuclear factor erythroid-2 related factor 2 (NRF2) in macrophages, LDL oxidative modification upregulates the mRNA expression of iron metabolism-associated genes (Hmox1 and Fpn) [86]. Iron retention further aggravates iron overload through the ox-LDL-mediated TLR4/NF-κB pathway in macrophages, due to cholesterol disequilibrium [9], implying that iron and lipid accumulation in macrophages within the atherosclerotic plaque synergistically promote AS. Iron chelators, such as desferrioxamine (DFO), decrease intracellular iron concentration by releasing free iron and lowering proinflammatory factor monocyte chemotactic protein-1 (MCP-1) and decreasing the macrophage markers, thereby delaying the development of aortic atherosclerotic lesion without the changes of total cholesterol and triglycerides in apoE^−/−^ mice serum [95]. Therefore, dietary iron intake restriction is a potential measure for inhibiting plaque formation, at least in part, by the reduction of iron deposition and LDL oxidation in vascular lesions [96]. Interestingly, iron deficiency upregulates atheroma inflammation and enhances the production of extracellular matrix metalloproteinase inducer (EMMPRIN)/matrix metalloproteinase-9 (MMP-9) from human monocyte-derived macrophages or foam cells by activating p38/ mitogen-activated protein kinase (MAPK)/NF-κB pathway [97], and by the overexpression of tumor necrosis factor-alpha (TNF-α) [98], interleukin-1β (IL-1β) [99], cyclooxygenase-2 [100], and prostaglandin E2 (PGE2) [100]. Iron overload and iron deficiency have been shown to activate pro-inflammatory responses. Iron overload induces ROS-related inflammation while iron deficiency regulates the expression of inflammatory factors at the transcription level. In conclusion, iron metabolic imbalance in macrophages accelerates the formation of AS via ferroptosis.

### The effects of ferroptosis in vascular smooth muscle cells on AS

The migration of vascular smooth muscle cells (VSMCs) into the intima is involved in the formation of initial atherosclerotic plaques [101, 102]. Upregulating the expression of GPx4 in VSMCs enhances artheroprotection by blocking oxidative stress and ROS [103]. Cigarette smoking is a common risk factor for AS [104]. A recent study reported that the cigarette smoke extract (CSE) significantly induced ferroptosis in VSMCs of rat, but not apoptosis or necroptosis [11]. Moreover, many atherosclerotic inflammatory factors, including interleukin-1β (IL-1β), IL-6, tumor necrosis factor-α (TNF-α), matrix metalloproteinase-2 (MMP-2), and MMP-9 were shown to be upregulated by CSE in the rat aortic smooth muscle cell line A7r5. However, vascular endothelial cells were not influenced by CSE [11]. We postulate that, compared to endothelial cells, VSMCs have different mechanisms for triggering ferroptosis, which may produce lipid peroxidation through different pathways.

## Future research directions of ferroptosis in AS field

Ferroptosis, a cell death mode characterized by iron-dependent lipid peroxidation, is an important intermediate link between the initial and advanced AS. Vascular endothelial ferroptosis accelerates AS. In addition, several AS-related pathophysiological events, including lipid and iron metabolism disorders, oxidative stress, and inflammatory responses, have been associated with ferroptosis. Furthermore, ferroptotic factors play a mutant role for AS (Table 1). The small molecules and drugs affecting the onset of ferroptosis also have an influence on AS (Table 2). However, the causal relationship and specific mechanisms should be further clarified.

**Table 1:** The effects of ferroptotic factors on AS.

Ferroptotic factors	Promote/inhibit	The effects on AS		References
Ferritin	Promote	Increase the thickness of carotid intima-media		[22]
TFR1	Promote	Accumulate in the nuclear regions of foamy cells		[25]
Hepcidin	Promote	Promote TLR4/NF-κB pathway to induce iron retention in murine macrophages		[9]
		Enhancing inflammation-mediated AS		[29]
		Knockout exhibited low iron levels in aortic macrophages and decreased aortic macrophage activities		[30]
ROS	Promote	ROS-induced phospholipid oxidation upregulated during I/R injury		[39]
15-LOX	Promote	Promoting ox-LDL deposition in the subendothelial space		[44]
		Changing intracellular cholesterol metabolism in macrophages		[46]
GPx4	Inhibit	Converting lipid hydroperoxides		[7]
ATF3	Inhibit	Inhibiting PI3K-MMP3 signal pathway, reducing elastic lamina damage, increasing plaque stability		[80]

**Table 2:** Small molecules and drugs that interfere with ferroptosis in AS-related cells.

Compound	Cell type	Treatment effects	References
Erastin	HUVEC	Induce ferroptosis with a rapid generation of ROS and a reduced viability	[76]
	MAEC	Induce ferroptosis with elevated ROS, lipid peroxidation, and MDA levels within the damaged mitochondria	[12]
RSL3	Macrophage	Induce ferroptosis associated with inducible NO synthase/NO•	[88]
Fer-1	HUVEC	Allevite ROS and maintain cell viability	[76]
	MAEC	Suppress the generation of peroxidation products	[12]
DFO	Macrophage	Decreasing the macrophage markers and lowering proinflammatory factor MCP-1 in apoE^−/−^ mice	[95]
Deferoxamine Mesylate	HUVEC	Rescued ferroptotic damage in endothelial cells	[77]
CSE	VSMC	Induced ferroptosis and upregulate atherosclerotic inflammatory factors	[11]

**Fig. 1:**
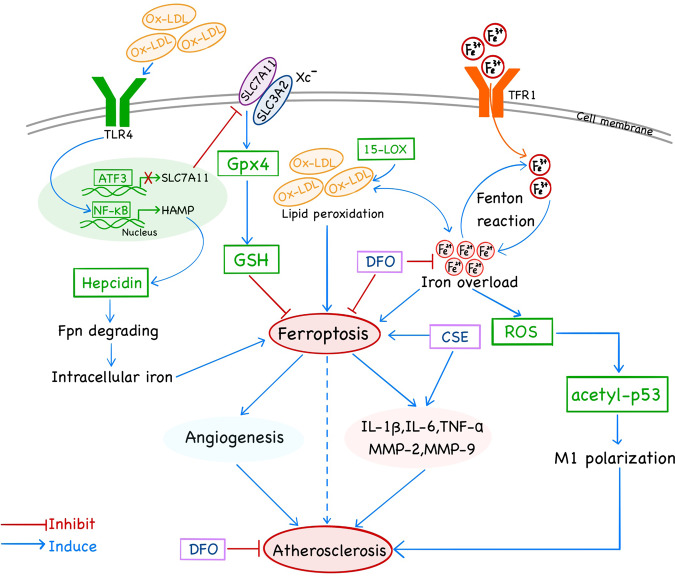
Molecular mechanisms and signaling pathways of ferroptosis interact with AS. Iron overload, along with ox-LDL as the major component of lipid peroxidation, are contribute to ferroptosis and promote AS, which can be transferred by DFO. Fe^3+^ through the cell membrane via TFR1, converted into Fe^2+^ by Fenton reaction. Too much Fe^2+^ causing iron overload in the cells, promote ferroptosis and accelerate the production of intracellular ROS, which promotes the polarization of M1 macrophages through the acetyl-p53 pathway to facilitate the occurrence of AS. The role of ox-LDL can be divided into two aspects. On the one hand, ox-LDL increases the expression of hepcidin by promoting the TLR4/NF-KB pathway, which enhances the degrading of Fpn, improving intracellular iron, and causing ferroptosis. On the other hand, the accumulation of intracellular lipid peroxidation has a mutual promotion effect with iron overload, which removal mainly through Gpx4. The interaction between ferroptosis and AS is probably through angiogenesis and inflammation.

Ferroptosis is a programmed cell death attributed to organelle and cytoplasmic membrane damage, in which the key hub is the overproduction and removal of ROS-induced lipid peroxides. The effects of lipid disorder-mediated ferroptosis on AS are multifaceted. In particular, hepcidin, ferritin, 15-LOX, and GPx4 are ferroptosis-related factors that mediate the production and clearance of lipid peroxides, which preliminarily are confirmed to be associated with AS. By accelerating Fenton reactions, intracellular iron transport proteins such as transferrin and transferrin receptors are highly associated with AS [25, 105]. Hepcidin overexpression increases intracellular iron concentration by degrading FPN proteins and subsequently, the presence of ferroptosis promotes the rate of AS-CVD [9, 29, 44, 86]. Furthermore, ox-LDL is involved in numerous factors of atherogenesis and its contribution to ferroptosis may be a novel mechanism for its damaging effects on AS. ox-LDL enhances iron overload which induces macrophage inflammation through the ROS/acetyl-p53 pathway [93, 94], while ox-LDL-induced TLR4/NF-κB activation mediates 15-LOX leading to AS-related cholesterol disequilibrium and inflammation. Compared to iron transport proteins that regulate lipid peroxides production, GPx4 is a crucial protein in the clearance of lipid peroxidation. GPx4 activation inhibits ferroptosis and alleviates AS injury [7] (Fig. 1). However, GPx4 knockdown reverses this effect. GPx4 should, therefore, be used to elucidate on the relationship between ferroptosis and AS to understand the specific role of lipid peroxidation in AS.

**Fig. 2:**
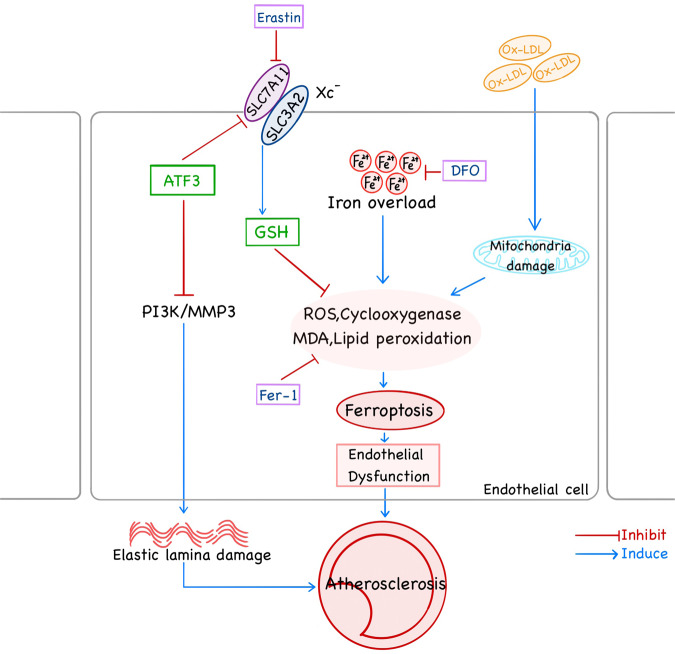
Role of ferroptosis mutual effect of AS in endothelial cells. The accumulation of lipid peroxides in endothelial cells that cause ferroptosis derived from two pathways. The extracellular ox-LDL causes endothelial cell mitochondrial damage, and intracellular iron overload causes lipid peroxide accumulation, which can be reserved by fer-1. Meanwhile, ATF3 inhibits its clearance pathway, and has a protective effect on atherosclerosis by reducing the damage of elastic membrane by inhibiting the PI3K-MMP3 pathway. The ferroptosis of endothelial cell and elastic lamina damage leads to endothelial dysfunction promoting the formation of AS.

Through AS, ferroptosis can occur in a variety of cells, such as macrophages, VSMCs, VECs. Ferroptosis promotes AS through lipid peroxidation-induced endothelial dysfunction (Fig. 2) [12]. Iron overload promotes M1 macrophage transformation through the ROS/acetyl-p53 pathway [94] and induces endothelial dysfunction by ferroptosis, which can be alleviated by DFO (Fig. 1) [12, 94]. However, it is unclear whether the direct exposure of VECs to serum iron would affect ferroptosis in endothelial cells and then raise the initiation of AS. In addition, in previous studies of AS, macrophages are the main inflammatory cells. Therefore, whether and how ferroptosis regulates macrophage inflammatory response that probably affects AS is noteworthy. Thus, it is conceivable that ferroptosis may play an important regulatory role in cell phenotype and function associated with AS.

Although several interesting discoveries have manifested some factors related to ferroptosis participate in the initiation and progression of AS, the exact roles and mechanisms remain to be further investigated. The below aspects deserve further research. Firstly, vitamin (Vit) E inhibits ferroptosis [106], and Vit E has been shown to increase antioxidant resistance in vitro and prevent atherosclerotic plaque formation [107], but a 4.5-year follow-up found no significant difference in the risk of cardiovascular events in patients with or without Vit E supplementation [108–110]. However, it is worth studying whether Vit E affects AS through ferroptosis. Secondly, that CSE can induce ferroptosis of VSMC, but not that of endothelial cells remains unknown [11], and it is speculated that there probably exist differences in the mechanism of ferroptosis between endothelial cells and VSMC. Thirdly, the specific mechanism of the effect of different macrophage phenotypes on ferroptosis has not been fully investigated.

In summary, studies on ferroptosis in the field of AS are still at a very early stage. The important role of ferroptosis in the occurrence and development of major chronic disease AS has attracted extensive attention. It is believed that more in-depth studies will explore and reveal the current limited molecular mechanism of ferroptosis, thus providing more enough scientific basis for the clinical application of targeting ferroptosis to the prevention and treatment of AS.
